# Steering Charge Kinetics of Tin Niobate Photocatalysts: Key Roles of Phase Structure and Electronic Structure

**DOI:** 10.1186/s11671-018-2578-2

**Published:** 2018-05-23

**Authors:** Shushu Huang, Chunyan Wang, Hao Sun, Xiaojing Wang, Yiguo Su

**Affiliations:** College of Chemistry and Chemical Engineering, Inner Mongolia University, Hohhot, 010021 Inner Mongolia People’s Republic of China

**Keywords:** Phase structure, Electronic structure, Charge separation, Charge transfer rate, Photocatalytic activity

## Abstract

Tin niobate photocatalysts with the phase structures of froodite (SnNb_2_O_6_) and pyrochlore (Sn_2_Nb_2_O_7_) were obtained by a facile solvothermal method in order to explore the impact of phase structure and electronic structure on the charge kinetics and photocatalytic performance. By employing tin niobate as a model compound, the effects of phase structure over electronic structure, photocatalytic activity toward methyl orange solution and hydrogen evolution were systematically investigated. It is found that the variation of phase structure from SnNb_2_O_6_ to Sn_2_Nb_2_O_7_ accompanied with modulation of particle size and band edge potentials that has great consequences on photocatalytic performance. In combination with the electrochemical impedance spectroscopy (EIS), transient photocurrent responses, transient absorption spectroscopy (TAS), and the analysis of the charge-carrier dynamics suggested that variation of electronic structure has great impacts on the charge separation and transfer rate of tin niobate photocatalysts and the subsequent photocatalytic performance. Moreover, the results of the X-ray photoelectron spectroscopy (XPS) indicated that the existent of Sn^4+^ species in Sn_2_Nb_2_O_7_ could result in a decrease in photocatalytic activity. Photocatalytic test demonstrated that the SnNb_2_O_6_ (froodite) catalyst possesses a higher photocatalytic activity toward MO degradation and H_2_ evolution compared with the sample of Sn_2_Nb_2_O_7_ (pyrochlore). On the basis of spin resonance measurement and trapping experiment, it is expected that photogenerated holes, O_2_^−•^, and OH^•^ active species dominate the photodegradation of methyl orange.

**Electronic supplementary material:**

The online version of this article (10.1186/s11671-018-2578-2) contains supplementary material, which is available to authorized users.

## Background

Energy and environment problems have limited the sustainable development of human society, with the enhancement of the utilization of fossil resources [1–3]. Photocatalysis based on semiconductors attracts great attention owning to its potential application in solving the global energy crisis and environmental pollution [4–7]. The light absorption and photogenerated charge carriers may be the mainly limitations of photocatalytic efficiency, both of which are intrinsically controlled by the electronic structure [8–11]. In this regard, research on engineering appropriate electronic structure of the photocatalyst showing controllable photocatalytic performance is emerged as a lucrative way to dissolve the above issues [12–14]. As we all know, that the electronic structure is dependent on the crystal structure of semiconductors in a degree [15]. A series of studies on the oxide semiconductors with different crystal structures, as well as their optimal photocatalytic performance, have been reported, such as NaTaO_3_/Na_2_Ta_2_O_6_, SrNb_2_O_6_/Sr_2_Nb_2_O_7_, Ba_5_Ta_4_O_15_/Ba_3_Ta_5_O_15_, and SrTa_2_O_6_/Sr_4_Ta_2_O_9_/Sr_5_Ta_4_O_15_ [16–20]. In principle, the detailed crystallographic and structural variations determine the native photocatalytic performance of semiconductors. To uncover the native structure-dependent properties, the investigation of the photocatalytic activity of photocatalysts with different crystal structures is fundamentally important.

Layered niobates and tantalates are regarded as the promising photocatalysts which are usually applied to the reaction of water splitting and the photodegradation of organic pollution [21]. Especially, tin niobate which exists in two crystal structures: the froodite (SnNb_2_O_6_) [22, 23] and the pyrochlore (Sn_2_Nb_2_O_7_) [24, 25] have attracted much attention for the visible light-responsive photocatalysts. Either SnNb_2_O_6_ or Sn_2_Nb_2_O_7_ exhibits close structural relations to many semiconductors. The identification of the structural variation is advantageous for regulation of photochemical and photophysical properties of tin niobate and other semiconductors. Foordite (SnNb_2_O_6_) as a typical 2D layered niobate semiconductor material where two corner-sharing NbO_6_ sheets linked together at their edges and a distorted SnO_8_ sheet due to the existence of a lone-pair electron is alternating [23, 26]. SnNb_2_O_6_ can be active under visible light irradiation because of narrow band-gap (~ 2.3 eV) [27]. Furthermore, as a result of the conduction band which consists of Nb 4d orbitals and the valence band which contains hybridized orbitals of Sn 5s and O 2p, a narrower band gap is observed in SnNb_2_O_6_ compared with other niobate compounds [28, 29]. Hence, the particular band structure results in SnNb_2_O_6_ being used as the photocatalysts for the hydrogen evolution reaction under visible light irradiation [27–31]. The pyrochlore oxides are different from foordite structure which has eight formula units of the general formula A_2_B_2_O_7_ in the cubic unit cell [32]. A three-dimensional network in corner-sharing tetrahedral was formed with A and B atoms individually, and O atoms were located around these atoms [33]. The valence band of Sn_2_Nb_2_O_7_ was composed with Sn 5s orbitals as same as that of SnNb_2_O_6_. Whereas, the differentiation of Sn/Nb molar ratio and phase structure cause the variation of conduction and valence band potentials between Sn_2_Nb_2_O_7_ and SnNb_2_O_6_ [28]. Although the pyrochlore structure has a similar band gap to that of froodite and the photocatalytic activity of these reported metal oxide photocatalysts does not seem high [34–36]. Therefore, both the phase structure and electronic structure may always play an important role in the photocatalytic performances. Hence, tin niobate photocatalysts with the phase structures of froodite (SnNb_2_O_6_) and pyrochlore (Sn_2_Nb_2_O_7_) were systematically investigated to uncover the nature of phase structure-dependent properties, including the size, shape, optical absorption, the activity of photo-induced carriers, and the photocatalytic activity.

In this work, a series of tin niobate photocatalysts were synthesized via a facile solvothermal method in order to explore the roles that phase structure and electronic structure played on the charge kinetics and photocatalytic performance. The characters of the obtained products, such as morphology, structure and optical/electric properties, were investigated systematically with various physicochemical techniques. The photocatalytic properties of the obtained photocatalysts were investigated by the photocatalytic hydrogen evolution reaction and the degradation of MO under visible light irradiation. Meanwhile, the photocatalytic reaction mechanism was proposed based on the exploration of actives species and ESR analysis over the obtained SnNb_2_O_6_ photocatalyst.

## Methods/Experimental

### Synthesis of SnNb_2_O_6_ and Sn_2_Nb_2_O_7_

K_7_HNb_3_O_19_•13H_2_O was obtained as precursor for the synthesis of photocatalysts. For the synthesis of the target materials, K_7_HNb_3_O_19_•13H_2_O (0.360 g) was dissolved into distilled water (8 mL) whose pH value was adjusted to 7 by adding HCl solution (2.4 mol•L^− 1^) under magnetic stirring. Meanwhile, the white suspension was formed. As SnCl_2_•2H_2_O (0.225 g) solution which was dissolved with deionized water (2 mL) adding into the above suspension, a yellow suspension was generated. Meanwhile, the pH value of this suspension was about 1. Moreover, the pH values (3, 5, 7, 9, and 11) of the above suspension were adjusted by 1 mol•L^− 1^ KOH under vigorous stirring. Then, the mixture was statically heated at 180 °C for 24 h. The resultant products were washed with deionized water consecutively and dried 12 h at 80 °C.

### Morphology, Structure, and Optical Properties Characterization

In order to study the crystal phase structures of the prepared samples, the wide-angle X-ray power diffraction (XRD) was used by performing on a Rigaku DMAX2500 X-ray diffractometer with Cu Kα radiation. Scanning electron microscopy (SEM) was performed on a HITACHI S-4800 apparatus, which applied to investigate the morphologies of the obtained photocatalysts. For the purpose of confirming the lattice spacing and the morphologies of the obtained samples, transmission electron microscopy (TEM) was recorded using a FEI Tecnai G^2^ F20 S-TWIN field emission microscope apparatus with an acceleration voltage of 200 kV. The ultraviolet-visible DRS of the samples were measured with a Perkin Elmer UV/VS/NIR Lambda 750 s spectrometer. The surface structures of the samples were measured by using Perkin Elmer IR spectrometer. The specific surface areas of the samples were measured on a Micromeritics ASAP 2020 Surface Area and Porosity Analyzer by Brunauer-Emmett-Teller (BET) technique. X-ray Photoelectron Spectroscopy (XPS) analyses were performed on an ESCALab220i-XL with a monochromatic Al Kα and charge neutralizer. The C 1 s peak at 284.6 eV was the referenced binding energy for all the samples. EPR spectra for superoxide radicals (sample, 4 mg; DMPO, 0.22 M; methanol solution volume, 2.0 mL) and hydroxyl radicals (sample, 4 mg; DMPO, 0.22 M; aqueous solution volume, 2.0 mL) were obtained on an ER200-SRC electron spin resonance spectrometer (Bruker, Germany) at 3186 G and 9056.895 MHz, which was performed in dark and visible light irradiation. Transient absorption spectroscopy (TAS) measurements were performed on a home-made setup equipped with a laser beam (532 nm, 1 mJ, 1 Hz), which was investigated in the range from 550 to 800 nm.

### Electrochemical Measurements

Electrochemical analysis was performed on a conventional three-electrode cell which was consisted in the working (the fabricated electrode), counter (a platinum wire), and reference electrodes (Ag/AgCl, 3M KCl). The synthesized photocatalysts powder was deposited on the FTO (F-doped tin oxide) glass by electrophoretic deposition. The depositional solution was composed by the acetone (50 mL) which contains photocatalysts power (40 mg) and iodine (10 mg). Two clean FTO glasses with a distance (2 cm) were immersed in the solution in parallel and a potential (20 V) was applied between the electrodes for 120 s using a DC power supply [37]. Electrochemical impedance spectroscopy (EIS) measurements were performed in the frequency from 0.1 Hz to 100 KHz, and the applied voltage was the open circuit voltage. The electrolyte was Na_2_SO_4_ aqueous solution (0.2 M, pH = 7) [38]. Mott-Schottky plot of SnNb_2_O_6_ was performed at the frequency of 1000 Hz in the dark.

### Evaluation of Photocatalytic Activity

Photocatalytic water splitting reactions were carried out by the suspension which was contained 0.1 g of the as-prepared photocatalyst, 80 mL deionized water, 20 mL triethanolamine (TEOA) which was the sacrificial electron donor. The reaction solution was evacuated several times to remove the air and ensure the reactor in an inert atmosphere before irradiation. A 300 W Xenon lamp with a filter (λ ≥ 420 nm) was used as light source in this photocatalytic system. The generated H_2_ gases were analyzed by an online gas chromatograph (GC2014C, TCD, Ar as the carrier).

The photocatalytic performance of semiconductors was measured using the photodegradation of methyl orange (MO) under visible light irradiation. Herein, the photocatalytic experimental procedure was as follows: the 50 mL reaction suspension was contained 2 × 10^− 5^ mol/L MO (50 mL), and the obtained photocatalysis (25 mg) which was continuously stirred for 2 h in dark to achieve the equilibrium of MO absorption/desorption on the sample surface before illumination. Then, the suspensions were irradiated by a 300 W mercury lamp with a filter (λ ≥ 420 nm). At given intervals, 5 mL solution was centrifuged, which was used to test the UV-vis absorption spectra by a UVIKON XL/XS Spectrometer.

## Results and Discussion

**Fig. 1:**
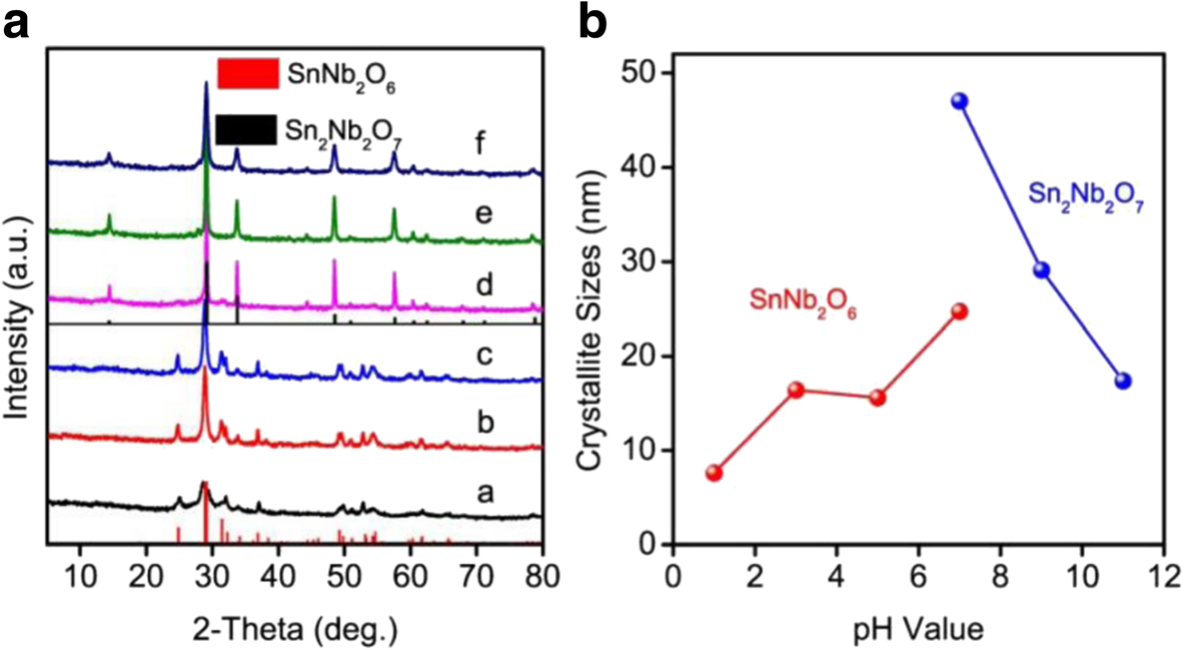
X-ray diffraction patterns of tin niobate prepared in different pH value of the reaction solution (1~ 11) (**a**). Vertical bars below the patterns represent the standard diffraction data from JCPDS files for SnNb_2_O_6_ (01-084-1810) and Sn_2_Nb_2_O_7_ (00-023-0593). Relationship between the crystallite sizes and pH value of the reaction solution (**b**)

Figure 1a shows the XRD patterns of the products obtained by hydrothermal method. The phases of the samples were changed with increase of pH values. It is clearly seen that the diffraction peaks concurred with the pure phase of monoclinic SnNb_2_O_6_ (JCPDS 01-048-1810) as the pH values were 1, 3, and 5. The sample obtained at pH = 7 was the mixed phases of SnNb_2_O_6_ and Sn_2_Nb_2_O_7_ which also can be observed from the SEM (Additional file 1: Figure S2). And the phases were the pure phase of Sn_2_Nb_2_O_7_ (JCPDS 00-023-0593) when the pH values were 9 and 11. And no impurity peaks from other phases can be detected. This may be ascribed to their different Sn precursors as the description in the previous report [39]. The change of the phase structure was also investigated via the infrared spectra (Additional file 1: Figure S1). The average crystallite sizes of the synthesized samples was calculated using the Debye-Scherrer formula *D* = kλ/βcosθ [40]. Figure 1b presents that the average particle sizes of the prepared photocatalysts were increased from 7.6 to 24.7 nm for the crystal structure of SnNb_2_O_6_ with increase of pH from 1 to 7 and decreased from 47.0 to 17.4 nm for Sn_2_Nb_2_O_7_ with the pH value up to 11. It is commonly recognized that materials possess a smaller particle size which always have a higher specific surface area and the better photocatalytic activity of the catalysts will be achieved, which can be further confirmed by the results of photocatalytic performance [41]. In addition, we found that the reaction temperature had an influence on the formation of pyrochlore (Sn_2_Nb_2_O_7_) as shown in Additional file 1: Figure S2.

**Fig. 2:**
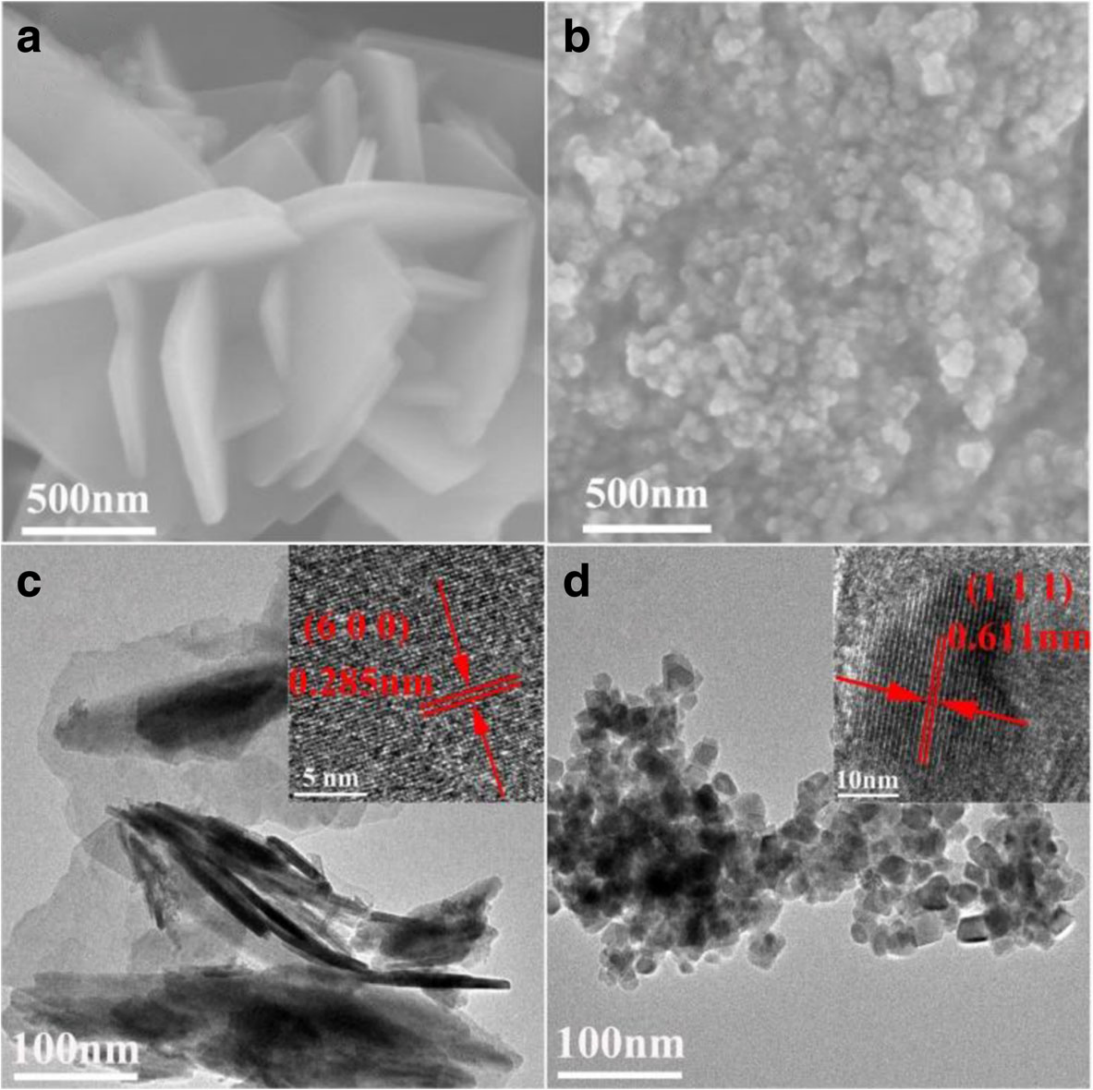
Typical SEM and TEM images of SnNb_2_O_6_ (**a**, **c**) and Sn_2_Nb_2_O_7_ (**b**, **d**).The insets are the HRTEM images of the samples

The morphologies and crystal structures of SnNb_2_O_6_ and Sn_2_Nb_2_O_7_ photocatalysts were investigated by field emission scanning electron microscopy (SEM) and transmission electron microscopy (TEM) (Fig. 2). It is clearly seen that the sample of SnNb_2_O_6_ was consisted of numerous irregular nanosheets (Fig. 2a, c) and the Sn_2_Nb_2_O_7_ was composed of uniform clumps (Fig. 2b, d). Meanwhile, the size of the clumps gradually decreased as the pH value increase (Additional file 1: Figure S3), which was consistent with the result of the average crystallite size in Fig. 1b. To identify the fine crystalline nature of the obtained samples, the high-resolution TEM was taken (inset images). As shown in Fig. 2c, the lattice plane space was about 0.285 nm corresponding to the (600) plane of SnNb_2_O_6_, and the lattice space of 0.611 nm was identical to the (111) plane of Sn_2_Nb_2_O_7_ illustrated in Fig. 2d.

**Fig. 3:**
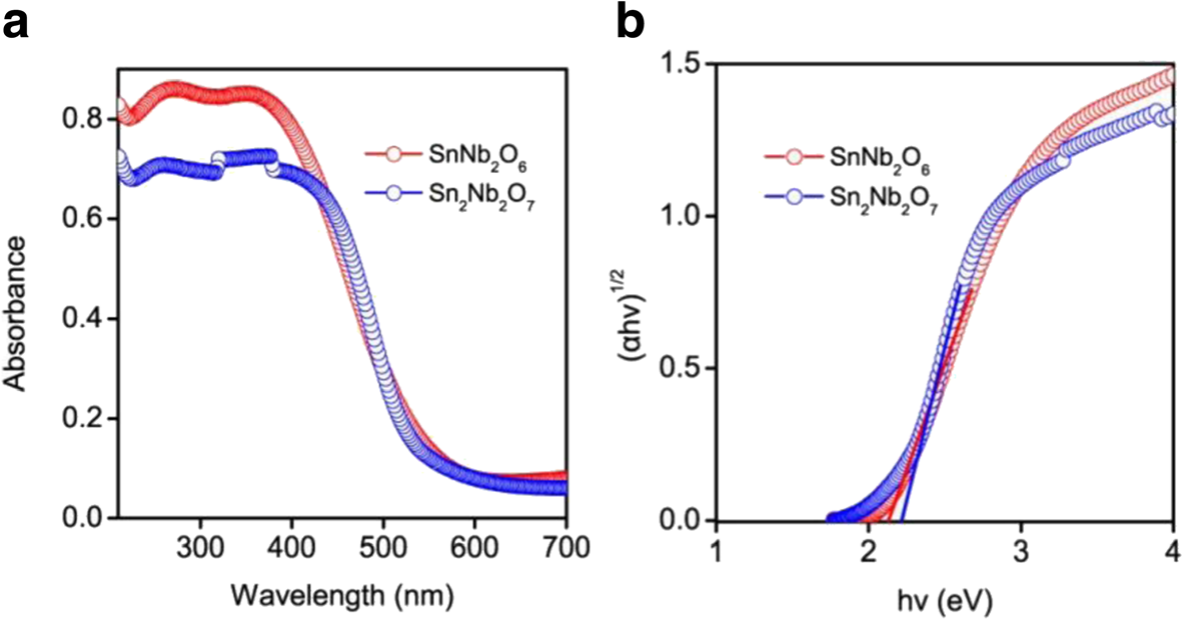
UV-visible diffuse reflectance spectra (**a**) and optical band gap (**b**) of the as-prepared SnNb_2_O_6_ and Sn_2_Nb_2_O_7_

In general, the band gap and energy level of a semiconductor are pivotal in determining the photocatalytic activity. It can be seen that all of the obtained samples had absorption in the visible region (Additional file 1: Figure S4). Meanwhile, the band gap transition from valence band to the conduction band was indicated by the steep edges in the DRS (Fig. 3a) [42]. The band gap energy *E*_*g*_ of the semiconductors (SnNb_2_O_6_ and Sn_2_Nb_2_O_7_) with an indirect electronic transition can be determined by the following equation: αhν = A (hν−*E*_g_)^1/2^, where α, ν, *E*_g_, and *A* are the absorption coefficient, incident light frequency, band gap, and constant, respectively [25, 43]. As illustrated in Fig. 3b, the band gap energy of Sn_2_Nb_2_O_7_ (2.22 eV) was larger than that of SnNb_2_O_6_ which was estimated to be ~ 2.12 eV. Meanwhile, the experimental data was closed to the calculated band gap of SnNb_2_O_6_ (~ 2.10 eV) which was different from Sn_2_Nb_2_O_7_ (~ 2.3 eV), and the difference between the band energies of the samples may be owing to the Sn:Nb ratio and the crystal structure were different from each other [28].

**Fig. 4:**
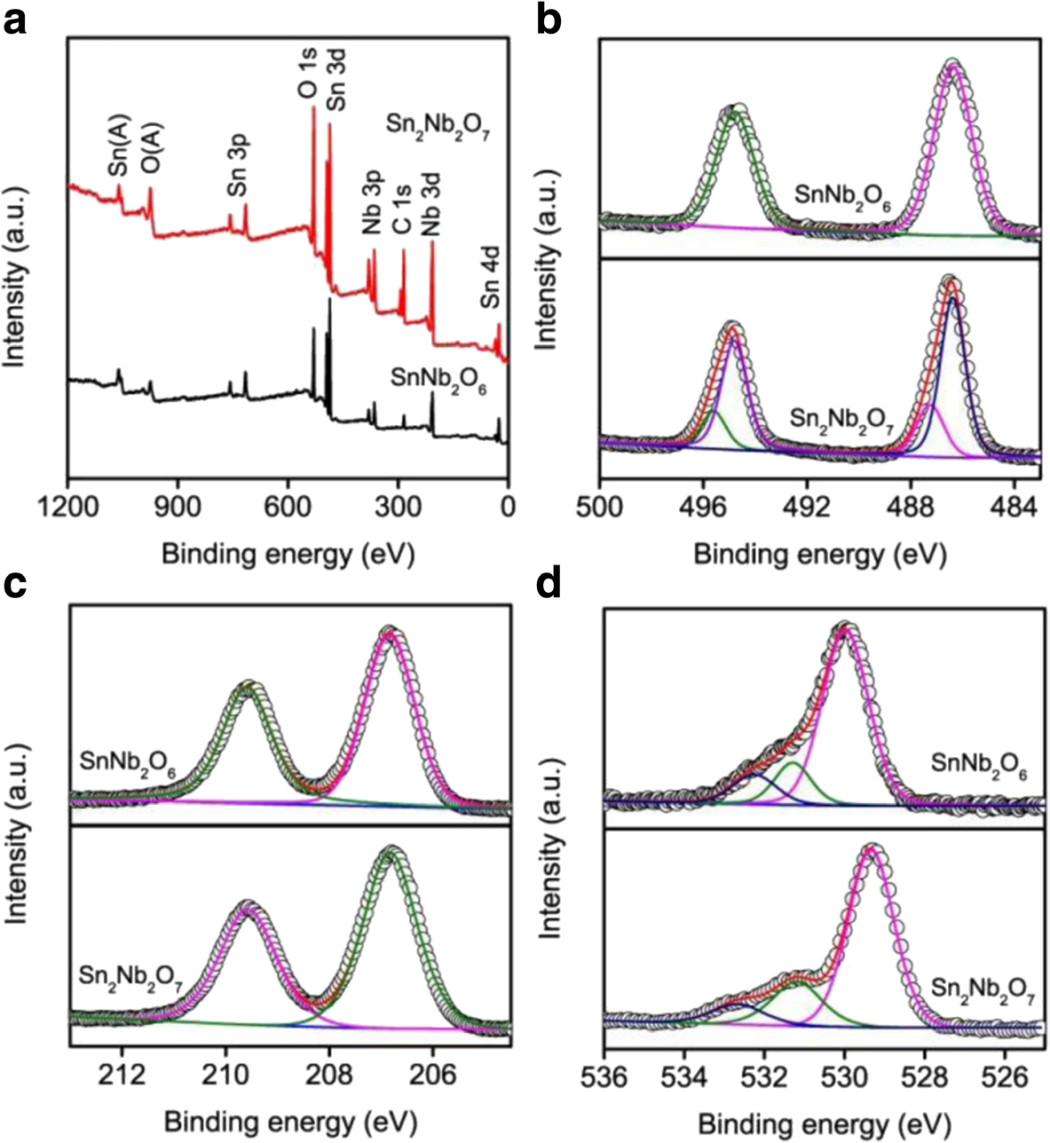
XPS spectra of SnNb_2_O_6_ and Sn_2_Nb_2_O_7_. **a** Survey spectrum, **b** Sn 3d, **c** Nb 3d, and **d** O 1s

The XPS measurement was performed to investigate the surface chemical compositions and chemical states of the photocatalysts. The survey XPS spectrum (Fig. 4a) of the as-prepared samples showed that Sn, Nb, O, and C were existed. The high-resolution XPS spectrum of Sn 3d (Fig. 4b) showed that Sn 3d XPS spectrum can be divided into two peaks with binding energies of ~ 486.4 and ~ 494.8 eV in SnNb_2_O_6_, which attributed to the Sn 3d_5/2_ and Sn 3d_3/2_ of Sn^2+^, respectively [44]. The binding energy of Sn 3d orbital for Sn_2_Nb_2_O_7_ exhibited four peaks as Sn^2+^ 3d_5/2_ at 486.4 eV, Sn^4+^ 3d_5/2_ at 487.2 eV, Sn^2+^ 3d_3/2_ at 494.8 eV, and Sn^4+^ 3d_3/2_ at 495.6 eV [45–48]. It indicated that Sn was present in the Sn^2+^ and Sn^4+^ chemical state on the surface of Sn_2_Nb_2_O_7_. The generation of the Sn^4+^ chemical state may be due to the oxidation of Sn^2+^ by the reaction system of strong alkalinity. As illustrated in Fig. 4c, the peaks located at ~ 206.9 and ~ 209.7 eV for all samples which correspond to the Nb 3d_5/2_ and Nb 3d_3/2_ and the structure splitting distance was about 2.8 eV, indicating that the Nb ions were existed in the form of Nb^5+^ [49]. Figure 4d illustrates XPS spectra of O 1s. We inferred that the variety of surface oxygen species was at least three kinds, on account of the three peaks in the O 1s spectrum for all the samples. The binding energy in O 1s spectra at about 530.1, 531.2, and 532.2 eV was assignable to the lattice oxygen (*O*_L_), surface hydroxyl groups (*O*_S_), and surface chemisorbed *O*_2_ which might be related to surface oxygen vacancies (*O*_ad_), respectively [50]. It is seen that the *O*_ad_ content in SnNb_2_O_6_ (11.8%) was higher than that in Sn_2_Nb_2_O_7_ (8.3%). Generally, a higher *O*_ad_ content implies a higher oxygen adsorption ability, which may expect a higher photocatalytic performance [51]. As a consequence, SnNb_2_O_6_ may display a greater photocatalytic activity than Sn_2_Nb_2_O_7_.

**Fig. 5:**
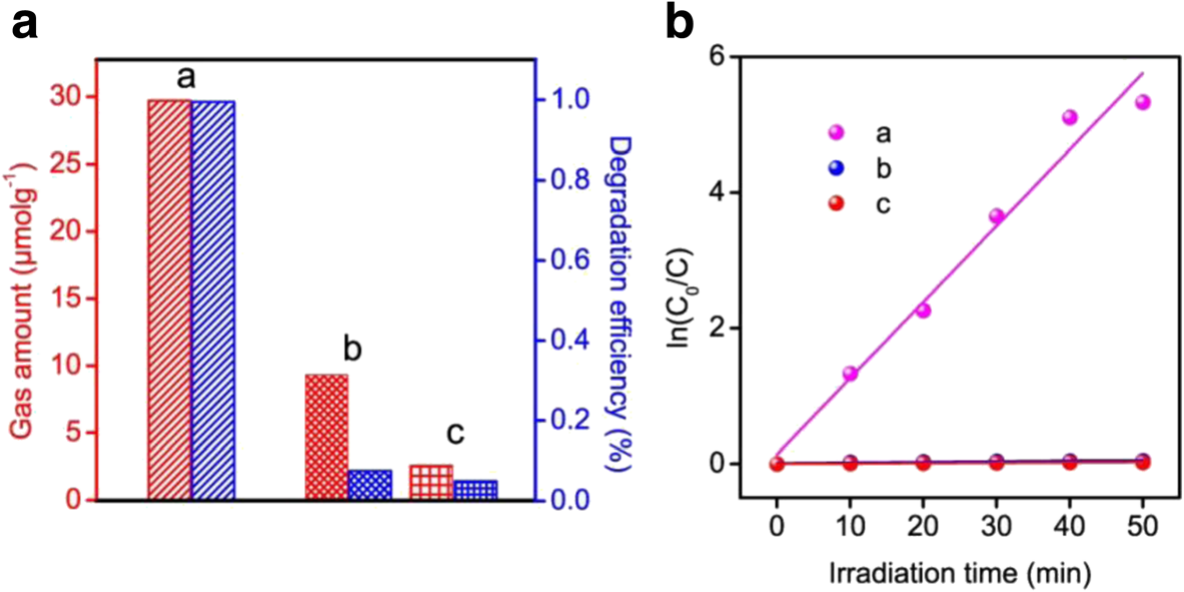
Photocatalytic water splitting over the samples. **a** SnNb_2_O_6_, **b** the mixed phases of SnNb_2_O_6_ and Sn_2_Nb_2_O_7_. **c** Sn_2_Nb_2_O_7_ in initial 5 h and MO degradation in the presence of the prepared photocatalyts in 70 min under visible light irradiation (**a**), relationship between ln (C_0_/C) and irradiation time for the degradation of MO over all samples (**b**)

In principle, different crystal structures shows difference on the photocatalytic activity, on account of their unique structure and electronic properties [16, 18–20]. The photocatalytic performance of the obtained samples was estimated by methyl orange (MO) decomposition as the model reaction. Before irradiation, all suspensions were stirred in dark for 120 min to ensure the establishment of adsorption/desorption equilibrium of methyl orange on the sample surfaces. Little adsorption of the MO molecules was observed for all samples. Moreover, the photodegradation of methyl orange in the absence of catalyst was also investigated. It is clearly showed that little change in the MO concentration was observed, which implied that visible light irradiation had little impact on the self-degradation of MO (Additional file 1: Figure S5). However, a continuous decrease of the characteristic absorption peaks of MO was observed as an addition of tin niobate samples under visible light irradiation (Additional file 1: Figure S5). As shown in Fig. 5a, all the tin niobate products had the photocatalytic performance toward the degradation of MO. Remarkably, SnNb_2_O_6_ obtained in the pH = 1 showed the highest photocatalytic activity with 99.6% degradation efficiency after illumination for 40 min. Meanwhile, with the increase of the pH value, the photocatalytic activity was highly decreased (Additional file 1: Figure S5). The curves of kinetics over different photocatalysts were shown in Fig. 5b. It is seen that there existed a linear relationship between the ln (C_0_/C) plot and the visible light irradiation time, suggesting a first-order kinetic reaction feature of methyl orange degradation [52]. And the SnNb_2_O_6_ possessed the maximal degradation rate constant (0.112 ± 0.008 min^− 1^). Moreover, the prepared samples also exhibited photocatalytic water splitting property under visible light irradiation. The photocatalytic H_2_ evolution activity of the as obtained samples had been evaluated from water in the presence of triethanolamine (TEOA) as a sacrificial electron donor and 1.0 wt.% Pt as co-catalytic to promote the H_2_ evolution activities. Additional file 1: Figure S6 presents the H_2_ evolution amount of the samples obtained at different pH value. The result revealed that the H_2_ evolution amount of SnNb_2_O_6_ prepared in pH = 1 was much higher than others. The optimal photocatalytic H_2_ evolution rate achieved for SnNb_2_O_6_ to be 5.94 μmol g^− 1^ h^− 1^, which was 3.2 and 11.4 times higher than that of the mixed phases of SnNb_2_O_6_ and Sn_2_Nb_2_O_7_ obtained in the pH value of 7 and Sn_2_Nb_2_O_7_ obtained in pH = 11 (Fig. 5a).

Having the aforementioned results in mind, it is highly necessary to explore the origination of the difference photocatalytic activity between the samples of SnNb_2_O_6_ and Sn_2_Nb_2_O_7_. As we all know, there basically exists three key steps in the overall potocatalysis process related to charge kinetics, i.e., generation, transfer, and consumption [53]. The first step is the generation of charge carriers which is mainly dominated by the energy band structure of the light-responding semiconductor. In order to maximize the number of photons converted into electron-hole pairs that participated in the photocatalytic process, the narrow band gap semiconductors for absorbing broader spectrum of solar energy was necessary. As mentioned above, the band gap energy of SnNb_2_O_6_ (2.12 eV) was smaller than Sn_2_Nb_2_O_7_ (2.22 eV). Furthermore, as shown in Fig. 4b, Sn presented the Sn^2+^ and Sn^4+^ chemical state on the surface of Sn_2_Nb_2_O_7_. As a result of the existent of Sn^4+^ ions, the photocatalytic activity was decreased, which could be attributed to the Nb^5+^ ions can be replaced with the Sn^4+^ ions and then a formed electron trap site by Sn^4+^ species was located below the conduction band [23, 28]. Hence, SnNb_2_O_6_ possessed of the advantage in the charge generation compared with Sn_2_Nb_2_O_7_ under visible light irradiation.

**Fig. 6:**
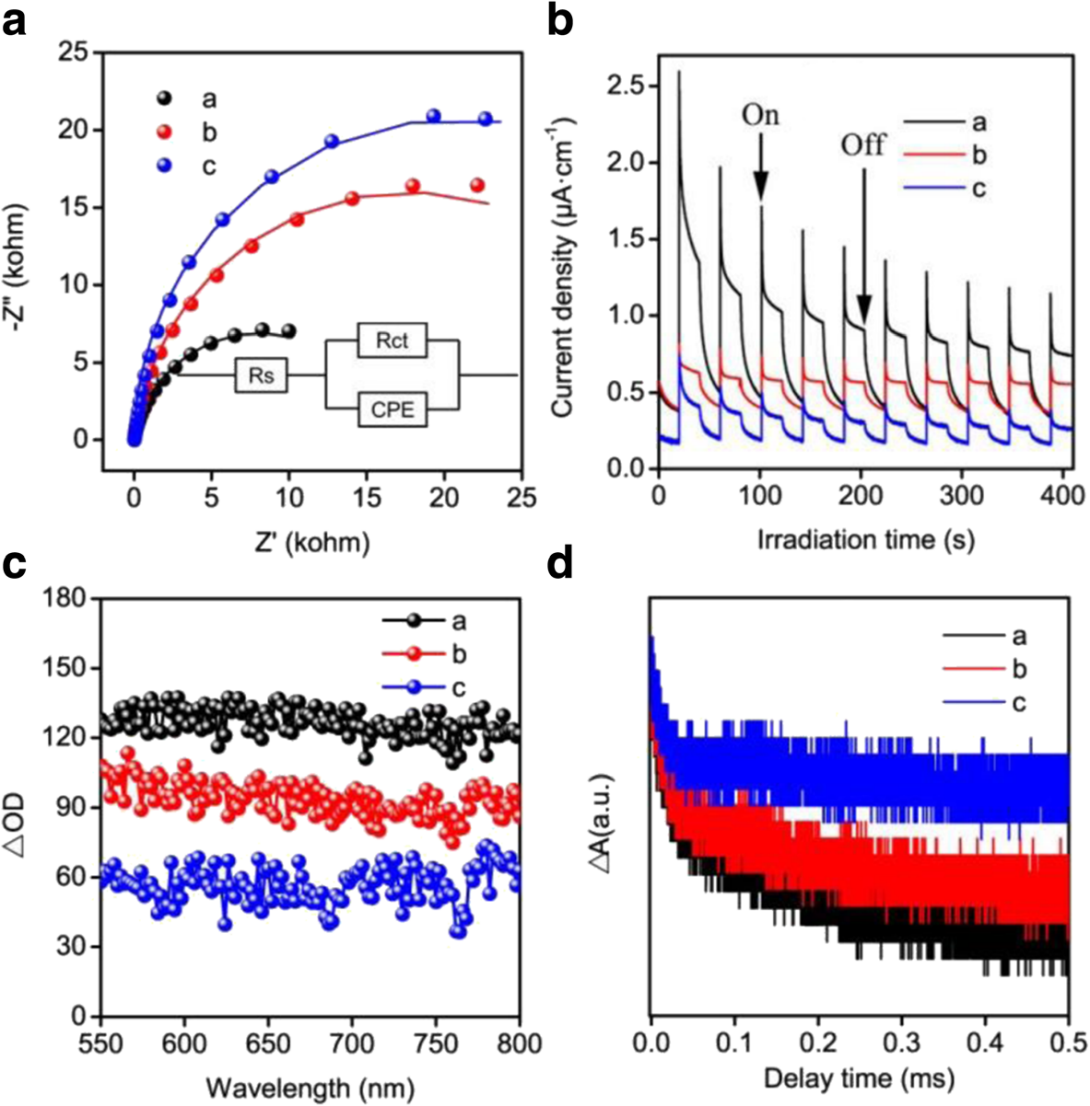
EIS Nyquist plots of a (SnNb_2_O_6_), b (the mixed phases of SnNb_2_O_6_ and Sn_2_Nb_2_O_7_), and c (Sn_2_Nb_2_O_7_) samples (**a**). Comparison of transient photocurrent response of the samples with light on/off cycles under white (neutral) light irradiation (LED 690 lm, [Na_2_SO_4_] = 0.2 M) (**b**). Transient absorption spectra measured at a delay time of 0.3 ms (**c**). Transient absorption decay kinetics of the prepared samples at an excitation wavelength of 600 nm (**d**)

Another most important step is the charge separation in the process of photocatalysis, which is the determination factor for the photocatalytic activity of a semiconductor, generally. Therefore, it is highly necessary to suppress detrimental electron-hole recombination during the charge-transfer. The electrochemical impedance spectroscopy (EIS) (Fig. 6a) was taken to investigate the charge-transfer resistance and separation efficiency of samples. The Nyquist plot data can be well-reproduced into solution-spreading resistance (*R*s), charge-transfer resistance (Rct) (inset of Fig. 6a) in parallel with a constant phase-element (CPE) [54, 55]. Rct for a (SnNb_2_O_6_), b (the mixed phases of SnNb_2_O_6_ and Sn_2_Nb_2_O_7_), and c (Sn_2_Nb_2_O_7_) samples were 16.1, 35.5, and 41.7 KΩ, respectively. The SnNb_2_O_6_ sample with the smallest Rct usually presented the lower resistance than others. According to the previous report, semiconductor own a smaller Rct which always achieved a higher separation efficiency of the photogenerated carriers and a faster transfer of interfacial charge during the photocatalytic process [56]. Furthermore, by way of transient photocurrent responses, we can give a profound understand for the separation efficiency and the transfer of photogenerated carriers. As shown in Fig. 6b, all of the samples exhibited prompt and reproducible photocurrent responses on each illumination. As observed, the transient photocurrent density of the SnNb_2_O_6_ was higher than others. In general, high photocurrent density typically indicated a stronger ability to promote electron shuttling and suppressing charge recombination, which eventually contributed to the enhancement in photocatalytic performance [57, 58]. Based on the analysis of the EIS and transient photocurrent response, the efficient charge separation and the improvement of electrical conductivity were achieved in SnNb_2_O_6_ compared with others, which may predict the enhancement of the photocatalytic performance.

Basically, the photocatalytic mechanism and the carrier dynamics also can be investigated by the time-resolved spectroscopy [59]. In order to further determine the excited state dynamics including charge separation, electron trapping and the recombination at materials surface, transient absorption spectroscopy (TAS) measurement was used [15]. As shown in Fig. 6c, all samples showed a broad and continuous absorption in the range of 550–800 nm with the excitation by pulse laser at 532 nm. According to previous literature, the broad absorption in visible light region could be ascribed to the effective separation of the photoinduced charges at different trap states in semiconductors [60–62]. As the transient absorption intensity at the same monitoring delay time and wavelength often represents the relative charge separation efficiency [63]. From Fig. 6c, it is clearly seen that the charge separation efficiency was highly improved in SnNb_2_O_6_ compared with the Sn_2_Nb_2_O_7_ sample. The home-made setup with an excitation wavelength of 600 nm was used to analyze the charge-carrier dynamics of the obtained samples. The results in Fig. 6d clearly suggested that a multi-exponential feature was exhibited from the decay curves for all samples. Furthermore, the effective time of the samples can be calculated according the previous research [64]. The effective lifetime τ for a (SnNb_2_O_6_), b (the mixed phases of the SnNb_2_O_6_ and Sn_2_Nb_2_O_7_), and c (Sn_2_Nb_2_O_7_) samples were 0.273, 0.271, and 0.264 ms, respectively. Clearly, the lifetime τ of the SnNb_2_O_6_ sample was larger than others. We all know that the longer lifetime always indicated the enhancement of the photogenerated electron-hole separation efficiency [65, 66]. Hence, the relatively long lived charge separation state of SnNb_2_O_6_ promised a higher charge separation efficiency and photocatalytic activity.

**Fig. 7:**
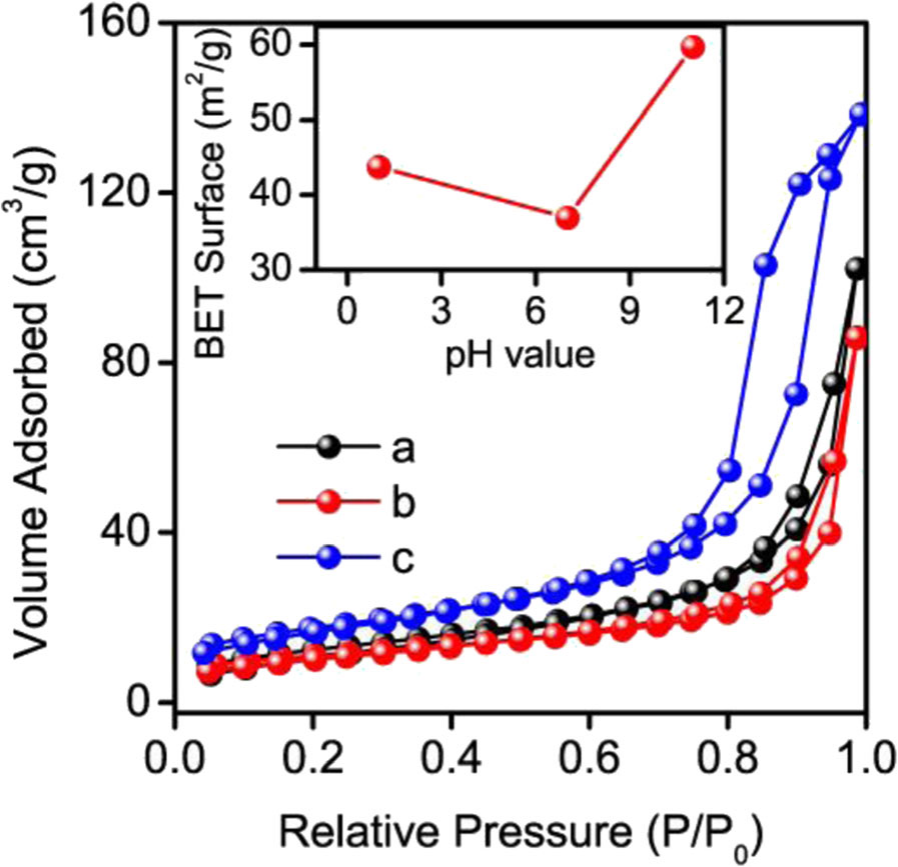
Nitrogen adsorption-desorption isotherms of the as-prepared samples (**a** SnNb_2_O_6_, **b** the mixed phases of SnNb_2_O_6_ and Sn_2_Nb_2_O_7_, and **c** Sn_2_Nb_2_O_7_). Inset figure shows the BET surface area as a function of pH value of the reaction solution

Usually, the enhancement of the surface adsorption and the increase of the active sites on the surface have very important impact on the charge-consumption step during the photocatalytic process. It has been commonly recognized that the surface active sites play a key role in the photocatalytic activity of semiconductor. Hence, the surface area may have an important influence on the photocatalytic performance of the prepared products. In general, larger surface area often possesses a higher photocatalytic activity because of the more active sites on the surface. The isotherm curves of the obtained samples showed a feature of type IV in the classification of Brunauer-Deming-Deming-Teller, as illustrated in Fig. 7. The BET surface area of a (SnNb_2_O_6_), b (the mixed phases of SnNb_2_O_6_ and Sn_2_Nb_2_O_7_), and Sn_2_Nb_2_O_7_ were 44, 37, and 60 m^2^/g, respectively (inset of Fig. 7). Obviously, the BET surface area of the SnNb_2_O_6_ was smaller than Sn_2_Nb_2_O_7_ and lager than the other one. Generally speaking, the samples with a smaller particle size always lead to higher specific surface area. However, the result of BET area was inconsistent with the particle size shown in Fig. 1b and the photocatalytic performance as shown in Fig. 5, which predicted the BET surface area had a minor impact on the photocatalytic performance of the semiconductors. Moreover, photocatalyst with a planar structure and a smaller size usually was beneficial to accelerating the transfer of photogenerated charge carriers from semiconductor interior to the reaction sites on surface and as a consequence the photocatalytic activity was improved [19, 67]. Thus, the SnNb_2_O_6_ sample which possessed the structure of nanosheets and the smallest average crystallite size shown in Fig. 1b had the superiority in the photocatalytic activity.

**Fig. 8:**
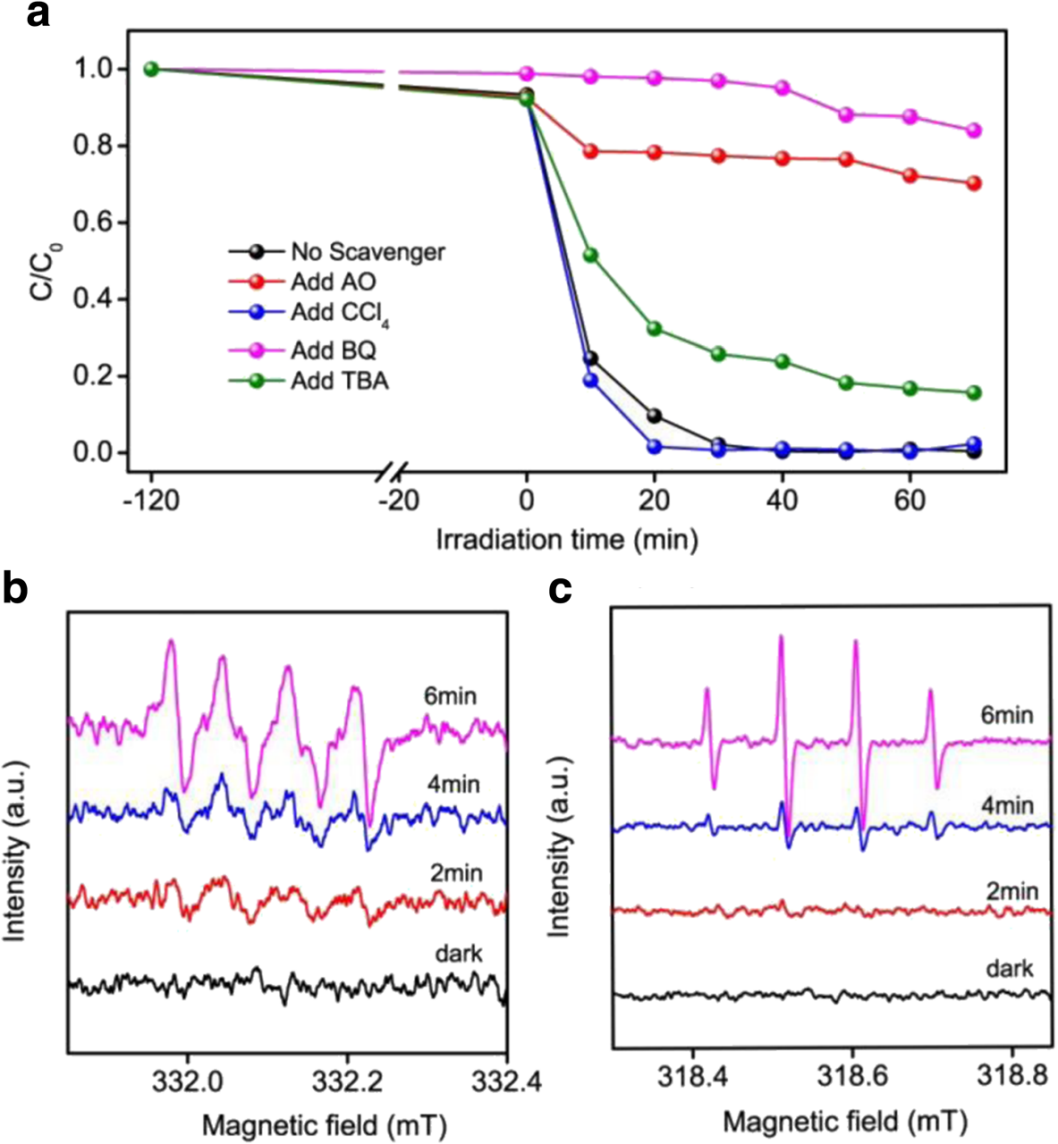
Effects of different scavengers on methyl orange degradation in the presence of SnNb_2_O_6_ under visible light irradiation (**a**). EPR spectra obtained from SnNb_2_O_6_ containing 0.22 M DMPO and 4.0 mg catalyst with total volume of 90% methanol/10% water (**b**) and 2 mL water (**c**) under different visible light irradiation time

As reported, it included four types of reactive species such as holes (h^+^), electron (e^−^), superoxide radicals (O_2_^−•^), and/or hydroxyl radicals (OH^•^) during the photocatalytic degradation of organic pollution [68]. In order to trace the effective radical species in the photocatalytic process, a series of controlled experiments by adding corresponding active species scavengers were carried out [69]. Briefly, 0.001 g of benzoquinone (BQ) was added to trap superoxide radical (O_2_^−•^), and 0.1 g of ammonium oxalate (AO) was added to trap hole (h^+^). Furthermore, the controlled experiments was proceeded by adding 2.5 mL carbon tetrachloride (CCl_4_) as an electron scavenger (e^−^) and 2.5 mL of tert-butyl alcohol (TBA) as a hydroxyl radical scavenger (OH^•^) [70, 71]. It was clear that the photodegradation rate of MO decreased significantly when TBA, BQ, and AO were added under visible light irradiation (Fig. 8a). Meanwhile, the photocatalytic activity was improved with the addition of CCl_4_. This may be due to the separation efficiency of photogenerated carriers that was enhanced with the addition of CCl_4_ as the electron scavenger, and then more holes and the corresponding active species were participated in the photocatalytic reaction, which would improve the degradation rate [72]. Based on the above result, the main active species in the photocatalytic decomposition of MO were included the oxidation reaction of the holes which generated in the valence and the formed O_2_^−•^ and OH^•^ on the surface of semiconductor. To further elucidate the actives involved in the photocatalytic process, electron paramagnetic resonance (EPR) technique was taken. 5, 5-Dimethyl-1-pyrroline-*N*-oxide (DMPO) was used as a spin trap to capture hydroxyl radical and superoxide species [73]. As shown in Fig. 8b, the characteristic EPR signal of DMPO-O_2_^−•^ was detected under the visible light irradiation and the intensity gradually increased with the increase of irradiation time. The result of the investigation of DMPO-OH^•^ adduct was presented in Fig. 8c which indicated that the active species of OH^•^ was generated in the process of photocatalytic under visible light irradiation and the signal increased with prolonged irradiation time. The formation of the OH^•^ and O_2_^−•^ active species in the process of the photocatalytic was determined by EPR technique. Meanwhile, the EPR analysis gave a direct evidence that the dominated active species during the photocatalytic decomposition MO were OH^•^ and O_2_^−•^.

The Mott-Schottky analysis was carried out to determine the flat band potential (*E*_fb_) and conduction band (CB) edges of the photocatalysts [74–76]. The positive slope was observed in the Schottky plots of all the products shown in Additional file 1: Figure S7 which demonstrated that the photocatalysts were assigned to n-type semiconductors [77, 78]. The flat band potentials (*E*_fb_) of the samples can be estimated using the extrapolation of the Mott-Schottky plot at the frequency of 1000 Hz and found to be − 0.685 eV for the SnNb_2_O_6_, − 0.67 eV for the mixed phases of SnNb_2_O_6_ and Sn_2_Nb_2_O_7_, and − 0.626 eV for the Sn_2_Nb_2_O_7_. It was known that the conduction band potentials of n-type semiconductors were closed to the flat potential [39, 79, 80]. Hence, the positions the conduction band of the prepared samples were − 0.685, − 0.67, and − 0.626 eV for SnNb_2_O_6_, the mixed phases of SnNb_2_O_6_ and Sn_2_Nb_2_O_7_, and the Sn_2_Nb_2_O_7_, respectively (inset of Additional file 1: Figure S6). From the results of the MS analysis, we can see that the variation of the phase structure from SnNb_2_O_6_ to Sn_2_Nb_2_O_7_ accompanied with the change of the band edge potentials. Meanwhile, the valence band of the sample can be evaluated by the band gap data (*E*_*g*_ = 2.12 eV) determined by the UV-vis spectra (Fig. 3), and the valence band of SnNb_2_O_6_ was 1.435 eV. This result was closed to previous reported results of the conduction band (− 0.68 eV) and valence band (1.42 eV) edge potentials of SnNb_2_O_6_ [43].

On the basis of the above experimental results, a possible photocatalytic mechanism was described as follows. For SnNb_2_O_6_, the conduction band (CB) and valence band (VB) edge potentials are − 0.685 and 1.435 eV, respectively. Under visible light irradiation, the photogenerated electrons were excited from the valence band to the conduction band of SnNb_2_O_6_ to reduction oxygen, while the photogenerated holes on the valence band of SnNb_2_O_6_ reacted with the contaminant and lead to the decomposition of methyl orange. The photogenerated electrons in the conduction band of SnNb_2_O_6_ reacted with electron acceptors including O_2_ existed in the system, leading to the formation of O_2_^−•^ active species and the subsequent degradation of methyl orange. The generated O_2_^−•^ radical species reacted with electrons in succession to produce active OH^•^, leading to the degradation of MO [81].

## Conclusions

In summary, we systematically investigated the tin niobate photocatalysts of SnNb_2_O_6_ (froodite) and Sn_2_Nb_2_O_7_ (pyrochlore) in order to uncover the impact of phase structure and electronic structure on the charge kinetics and the subsequent improvement of photocatalytic activity. The band gap was changed with the transformation of phase structure, which contributed to the advantage for SnNb_2_O_6_ in charge generation compared with Sn_2_Nb_2_O_7_. The existent of Sn^4+^ in Sn_2_Nb_2_O_7_ resulted in a decrease in the photocatalytic activity, because part of the Nb^5+^ ions can be replaced with Sn^4+^ ions in tin niobates, and the Sn^4+^ species formed an electron trap site which located below the conduction band. On the other hand, the efficient charge separation, the reduction of resistance, and the improvement of charge transfer rate, which dramatically enhanced the photocatalytic activity toward water reduction and MO degradation. The optimal photocatalytic activity toward H_2_ evolution of SnNb_2_O_6_ showed 11.4 times improvement with respect to that of the Sn_2_Nb_2_O_7_. Meanwhile, the SnNb_2_O_6_ possessed the maximal degradation rate constant (0.112 ± 0.008 min^− 1^). Additionally, the quenching effects of different scavengers suggested that the dominated active species in the photodegradation reaction were holes, O_2_^−•^, and OH^•^.

## Additional file

Additional file 1: **Figure S1.** FT-IR spectra of the as-prepared photocatalysts. **Figure S2.** X-ray diffraction patterns of tin niobate prepared under different reaction temperature of 140°C (a), 160°C (b), 180°C (c), and 200°C (d). **Figure S3.** Typical SEM images of photocatalysts prepared at different pH values (1~ 11) of the reaction solution. **Figure S4.** UV-visible diffuse reflectance spectra of the as-prepared photocatalysts. **Figure S5.** Normalized concentration of methyl orange (MO) versus visible light irradiation time in the presence of as-prepared photocatalysts. **Figure S6.** The time course of photocatalytic H_2_ evolution of all prepared samples under visible light irradiation (λ ≥ 420) by using TEOA as sacrificial agent and 1.0 wt.% of Pt as cocatalyst. **Figure S7.** Mott-Schottky plots of the samples collected at the frequency of 1000 Hz. (DOC 2510 kb)

## Abbreviations

BET: Brunauer-Emmett-Teller
DRS: Diffuse reflection spectroscopy
EIS: Electrochemical impedance spectroscopy
EPR: Electron paramagnetic resonance
FTO: Fluorine-doped tin oxide
SEM: Scanning electron microscopy
TAS: Transient absorption spectroscopy
TEM: Transmission electron microscopy
XPS: X-ray photoelectron spectroscopy
XRD: X-ray power diffraction
