# Supplementary material for: Steering Charge Kinetics of Tin Niobate Photocatalysts: Key Roles of Phase Structure and Electronic Structure
Source: Nanoscale Res Lett. 2018 May 23;13:161. doi: 10.1186/s11671-018-2578-2 (PMC5966348; doi:10.1186/s11671-018-2578-2)
Supplement: Supplementary file 1 — Figure S1. FT-IR spectra of the as-prepared photocatalysts. Figure S2. X-ray diffraction patterns of tin niobate prepared under different reaction temperature of 140°C (a), 160°C (b), 180°C (c), and 200°C (d). Figure S3. Typical SEM images of photocatalysts prepared at different pH values (1~ 11) of the reaction solution. Figure S4. UV-visible diffuse reflectance spectra of the as-prepared photocatalysts. Figure S5. Normalized concentration of methyl orange (MO) versus visible light irradiation time in the presence of as-prepared photocatalysts. Figure S6. The time course of photocatalytic H2 evolution of all prepared samples under visible light irradiation (λ ≥ 420) by using TEOA as sacrificial agent and 1.0 wt.% of Pt as cocatalyst. Figure S7. Mott-Schottky plots of the samples collected at the frequency of 1000 Hz. (DOC 2510 kb) [file 11671_2018_2578_MOESM1_ESM.doc]

Additional file 1

Steering charge kinetics of tin niobate photocatalysts: Key roles of phase structure and electronic structure

Shushu Huang, Chunyan Wang, Hao Sun, Xiaojing Wang, Yiguo Su*

Shushu Huang (huangxiaoya1234@126.com) Chunyan Wang (747954727@qq.com) Hao Sun (1126068055@qq.com) Xiaojing Wang (Wang_xiao_jing@hotmail.com)

Yiguo Su (cesyg@imu.edu.cn) (corresponding author)

College of Chemistry and Chemical Engineering, Inner Mongolia University, Hohhot, Inner Mongolia 010021, P. R. China

* Fax: +86-471-4992981; Tel: +86-471-4344579;

Fig. S1 FT-IR spectra of the as-prepared photocatalysts.


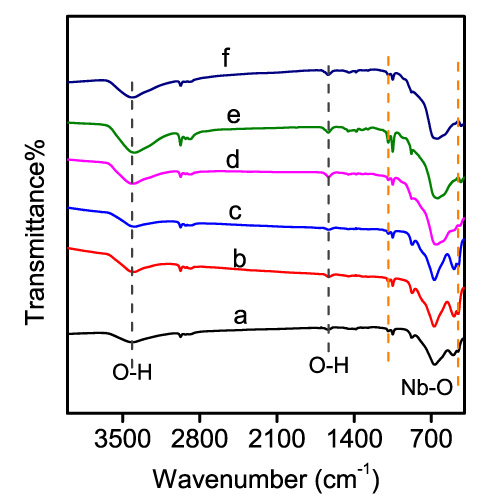


Chemical species adsorbed on the surfaces of the as-prepared samples were investigated by infrared spectra. As indicated in Fig.S1, all samples showed similar infrared spectra. The broad absorption band appeared at about 3400 cm^-1^ and 1630 cm^-1^ can be ascribed to the typical vibration of O-H of surface H_2_O layers . A series of intense absorption peaks from ~1100 to 450 cm^-1^ are related to the typical Nb-O vibration in the NbO_6_ octahedron . However, it is clearly seen that the two absorption peaks located at 500 cm^-1^ and 450 cm^-1^ were disappeared and the line width of the Nb-O vibration at 671 cm^-1^ was greatly broadened when the reaction solution is alkaline. This observation is also related to the formation of Sn_2_Nb_2_O_7_ with the increasing of the pH value of reaction solution. It is corresponded with the result of the XRD (Fig.1a).

Fig. S2 X-ray diffraction patterns of tin niobate prepared under different reaction temperature of 140℃ (a), 160℃ (b), 180℃ (c) and 200℃ (d).


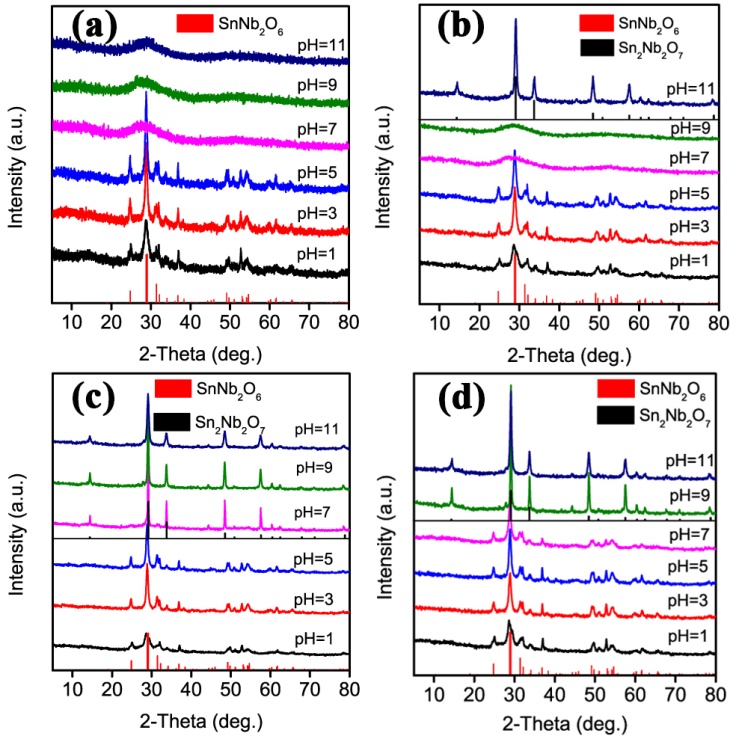


As shown in Fig. S2, it is seen that the pure phase of SnNb_2_O_6_ was obtained as the pH values were 1, 3 and 5 when the reaction temperature were 140℃, 160℃, 180℃ and 200℃. Whereas, the amorphous samples were obtained as the pH values were 7, 9 and 11 under the temperature of 140℃. And that the amorphous samples were also gained as the pH values were 7 and 9 under the temperature of 160℃. The sample of Sn_2_Nb_2_O_7_ was received as pH = 9, 11 under 180℃ and 200℃ and pH = 11 under 160℃. As mentioned above, we can conclude that the reaction temperature had non-impact on the formation of SnNb_2_O_6_, nevertheless, the pH value of solution and reaction temperature both had important influence on the formation of Sn_2_Nb_2_O_7_.

Fig. S3 Typical SEM images of photocatalysts prepared at different pH values (1~11) of the reaction solution.


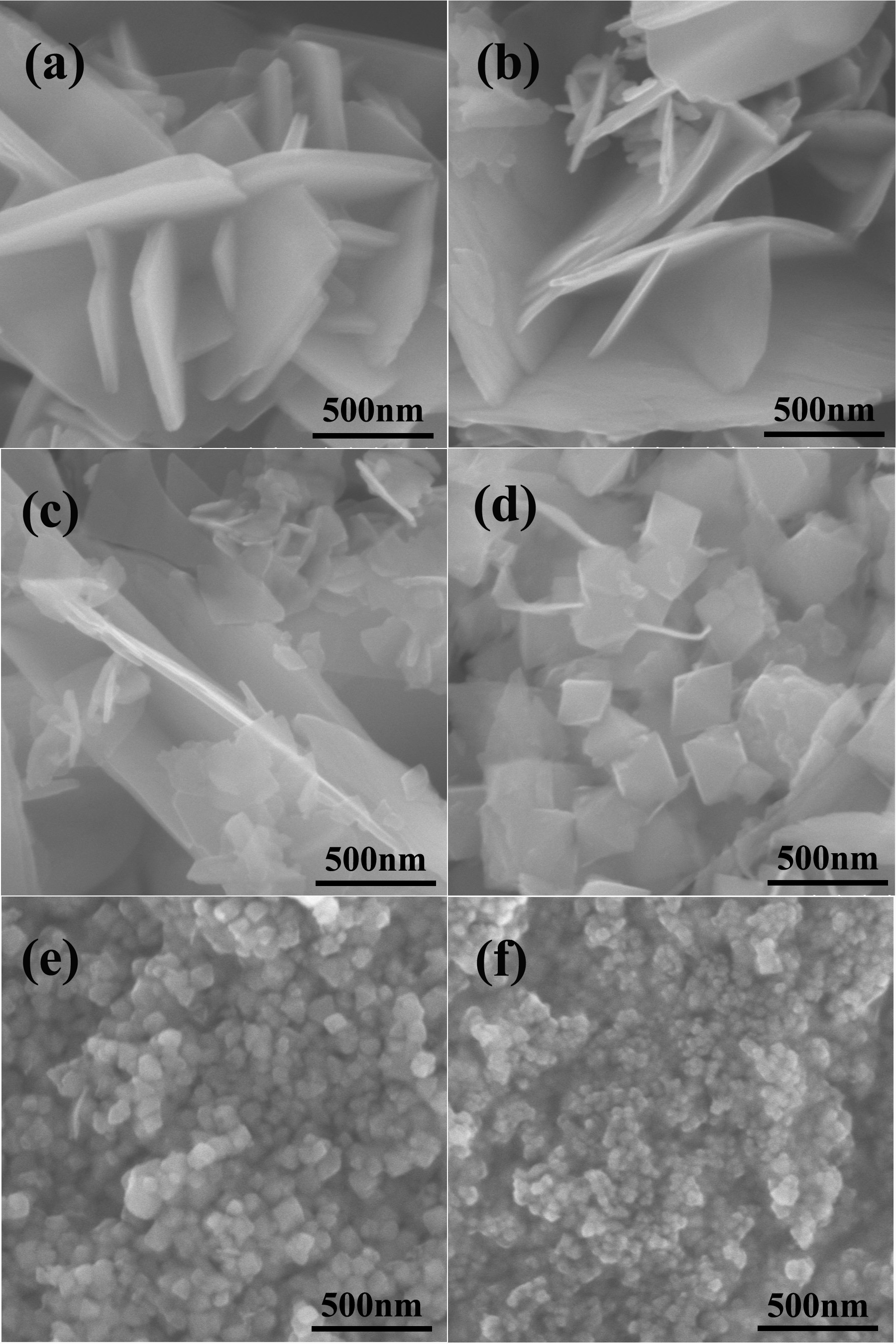


Fig. S4 UV-visible diffuse reflectance spectra of the as-prepared photocatalysts.


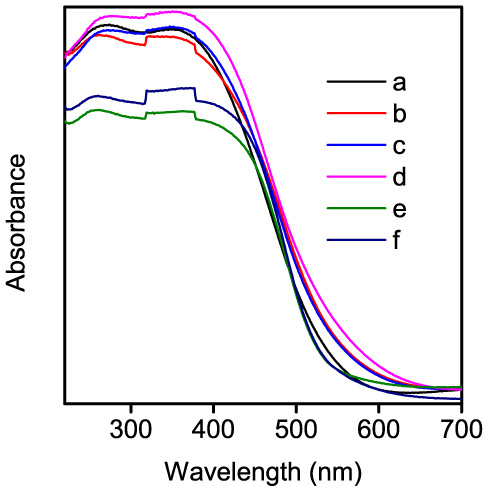


Fig. S5 Normalized concentration of methyl orange (MO) versus visible light irradiation time in the presence of as-prepared photocatalysts.


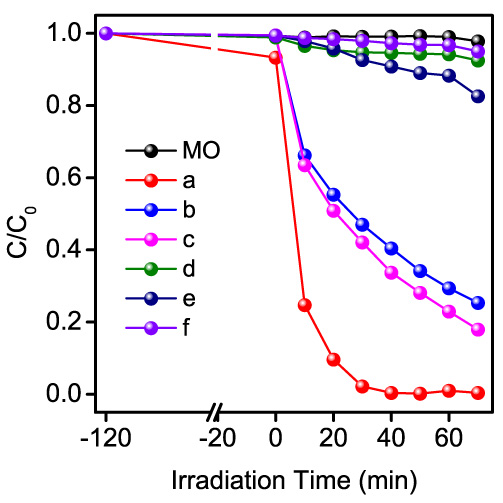


Fig. S6 The time course of photocatalytic H_2_ evolution of all prepared samples under visible light irradiation (λ ≥ 420) by using TEOA as sacrificial agent and 1.0 wt. % of Pt as cocatalyst.


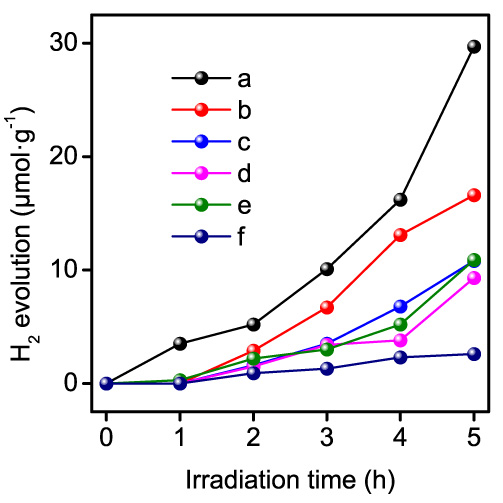


Fig. S7 Mott-Schottky plots of the samples collected at the frequency of 1000Hz.


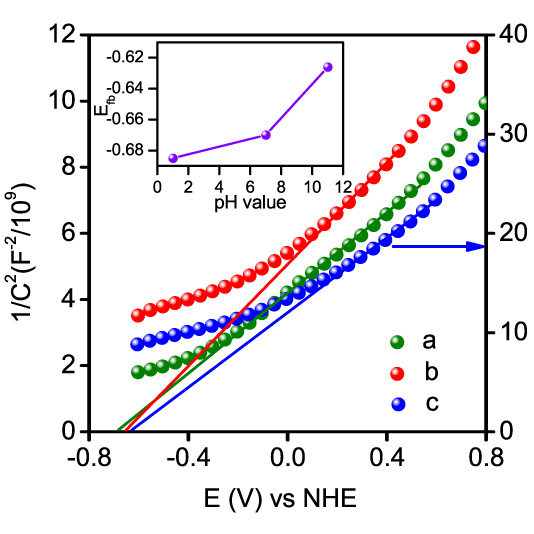


**References**

1. Su Y, Li G, Wang X, Li L (2012) Bridging between structure and optimum luminescence for nearly monodispersed Ce_1−x_Gd_x_F_3_:Eu^3+^ nanoparticles. J Lumin 132: 1407-1413

2. Shanker V, Samal SL, Pradhan GK, Narayana C, Ganguli AK (2009) Nanocrystalline NaNbO_3_ and NaTaO_3_: Rietveld studies, Raman spectroscopy and dielectric properties. Solid State Sci 11: 562-569

3. Su Y, Peng L, Guo J, Huang S, Lv L, Wang X (2014) Tunable Optical and Photocatalytic Performance Promoted by Nonstoichiometric Control and Site-Selective Codoping of Trivalent Ions in NaTaO_3_. J Phys Chem C 118: 10728-10739

4. Lavat AE, Baran EJ (2003) IR-spectroscopic characterization of A_2_BB’O_6_ perovskites. Vibrational Spectroscopy 32: 167-174
